# Comparative Effectiveness of Machine Learning Approaches for Predicting Gastrointestinal Bleeds in Patients Receiving Antithrombotic Treatment

**DOI:** 10.1001/jamanetworkopen.2021.10703

**Published:** 2021-05-21

**Authors:** Jeph Herrin, Neena S. Abraham, Xiaoxi Yao, Peter A. Noseworthy, Jonathan Inselman, Nilay D. Shah, Che Ngufor

**Affiliations:** 1 Division of Cardiology, Yale School of Medicine, New Haven, Connecticut; 2 Division of Gastroenterology and Hepatology, Department of Medicine, Mayo Clinic, Scottsdale, Arizona; 3 Robert D. and Patricia E. Kern Center for the Science of Health Care Delivery, Mayo Clinic, Rochester, Minnesota; 4 Division of Health Care Delivery Research, Mayo Clinic, Rochester, Minnesota; 5 Department of Cardiovascular Medicine, Mayo Clinic, Rochester, Minnesota; 6 OptumLabs, Cambridge, Massachusetts; 7 Department of Artificial Intelligence and Informatics, Mayo Clinic, Rochester, Minnesota

## Abstract

**Importance:**

Anticipating the risk of gastrointestinal bleeding (GIB) when initiating antithrombotic treatment (oral antiplatelets or anticoagulants) is limited by existing risk prediction models. Machine learning algorithms may result in superior predictive models to aid in clinical decision-making.

**Objective:**

To compare the performance of 3 machine learning approaches with the commonly used HAS-BLED (hypertension, abnormal kidney and liver function, stroke, bleeding, labile international normalized ratio, older age, and drug or alcohol use) risk score in predicting antithrombotic-related GIB.

**Design, Setting, and Participants:**

This retrospective cross-sectional study used data from the OptumLabs Data Warehouse, which contains medical and pharmacy claims on privately insured patients and Medicare Advantage enrollees in the US. The study cohort included patients 18 years or older with a history of atrial fibrillation, ischemic heart disease, or venous thromboembolism who were prescribed oral anticoagulant and/or thienopyridine antiplatelet agents between January 1, 2016, and December 31, 2019.

**Exposures:**

A cohort of patients prescribed oral anticoagulant and thienopyridine antiplatelet agents was divided into development and validation cohorts based on date of index prescription. The development cohort was used to train 3 machine learning models to predict GIB at 6 and 12 months: regularized Cox proportional hazards regression (RegCox), random survival forests (RSF), and extreme gradient boosting (XGBoost).

**Main Outcomes and Measures:**

The performance of the models for predicting GIB in the validation cohort, evaluated using the area under the receiver operating characteristic curve (AUC), sensitivity, specificity, positive predictive value, and prediction density plots. Relative importance scores were used to identify the variables that were most influential in the top-performing machine learning model.

**Results:**

In the entire study cohort of 306 463 patients, 166 177 (54.2%) were male, 193 648 (63.2%) were White, the mean (SD) age was 69.0 (12.6) years, and 12 322 (4.0%) had experienced a GIB. In the validation data set, the HAS-BLED model had an AUC of 0.60 for predicting GIB at 6 months and 0.59 at 12 months. The RegCox model performed the best in the validation set, with an AUC of 0.67 at 6 months and 0.66 at 12 months. XGBoost was similar, with AUCs of 0.67 at 6 months and 0.66 at 12 months, whereas for RSF, AUCs were 0.62 at 6 months and 0.60 at 12 months. The variables with the highest importance scores in the RegCox model were prior GI bleed (importance score, 0.72); atrial fibrillation, ischemic heart disease, and venous thromboembolism combined (importance score, 0.38); and use of gastroprotective agents (importance score, 0.32).

**Conclusions and Relevance:**

In this cross-sectional study, the machine learning models examined showed similar performance in identifying patients at high risk for GIB after being prescribed antithrombotic agents. Two models (RegCox and XGBoost) performed modestly better than the HAS-BLED score. A prospective evaluation of the RegCox model compared with HAS-BLED may provide a better understanding of the clinical impact of improved performance.

## Key Points

**Question:**

Does machine learning predict gastrointestinal bleeding (GIB) better than existing risk models in patients who use antithrombotic drugs?

**Findings:**

In this cross-sectional study of 306 463 patients, 2 machine learning approaches, regularized Cox proportional hazards regression and extreme gradient boosting, better predicted GIB risk than the most widely used existing risk model; a third approach, random survival forests, performed similarly to the existing model.

**Meaning:**

A prospective evaluation of the best-performing model may improve understanding of the clinical impact of using machine learning to predict the risk of GIB in patients who use antithrombotic drugs.

## Teaser

This cross-sectional study compares 3 machine learning algorithms with the most widely used existing model to assess which best predicts the risk of gastrointestinal (GI) bleeding in adult patients receiving antithrombotic treatment.

## Introduction

Physicians have long used prediction models to stratify patients according to their risk of adverse outcomes. Such risk stratification can promote better treatment decisions, more efficient use of monitoring, and better implementation of approaches to mitigate risk.^1^ One outcome for which risk stratification is routinely used is related to the decision to prescribe antithrombotic medications (vitamin K antagonists, direct oral anticoagulants [DOACs], and/or thienopyridine antiplatelet agents) to patients with cardiovascular diseases. One of the key goals of this risk stratification is to incorporate the risk of gastrointestinal bleeding (GIB) in the context of the treatment decision. Given the severity of GIB^2,3^ and the widespread use of antithrombotic agents in this population, several risk models have been developed to predict bleeding, including HAS-BLED (hypertension, abnormal kidney and liver function, stroke, bleeding, labile international normalized ratio, older age, and drug or alcohol use), ATRIA (anticoagulation and risk factors in atrial fibrillation), ORBIT (Outcomes Registry for Better Informed Treatment of Atrial Fibrillation), and HEMORR(2)HAGES (hepatic or kidney disease, ethanol abuse, malignancy, older age, reduced platelet count or function, rebleeding, hypertension, anemia, genetic factors, excessive fall risk, and stroke).^4,5,6,7^

Of these models, HAS-BLED has demonstrated the best performance among these scores, with an area under the receiver operating characteristic (ROC) curve (AUC) of 0.68 in a real-world population.^4^ All of the models are subject to important limitations, including model development using data sets with only 50 to 150 annual GIB events, lack of inclusion of more contemporary medications (namely DOACs and second-generation antiplatelet agents), and lack of revision to reflect medical advances, which may lead to a temporal lag in the models and outcomes as new technologies and clinical practices evolve. For example, the HAS-BLED score was derived to predict major bleeding in patients treated with warfarin, yet clinical extrapolation to estimate the risk of GIB is common. The HAS-BLED score may not accurately reflect the GIB risk in contemporary practice that has expanded to include DOACs and second-generation thienopyridine antiplatelet agents—drugs that are often used in combination. Furthermore, the HAS-BLED model may underestimate GIB in some patients, including older patients with multiple comorbidities.^8,9,10^

Given that the performance and utility of prediction models depends on the data source and methods used, we hypothesized that machine learning approaches applied to a larger, more recent data set with a broader range of parameters might produce a risk model with superior performance characteristics than those of HAS-BLED. Many machine learning approaches previously have been found to perform markedly better than risk models based on conventional approaches. This is especially true for rare outcomes, such as GIB (which occurs in 5% of patients aged <75 years who receive anticoagulant or antithrombotic monotherapy^11^), for which typical data sets may contain few events and small absolute differences in detecting true-positive cases can result in low sensitivity. Therefore, we aimed to test the ability of 3 machine learning algorithms to predict GIB in patients with atrial fibrillation, ischemic heart disease, or venous thromboembolism who recently started antithrombotic treatment.

## Methods

This cross-sectional study was exempted from the need for approval and informed consent by the Mayo Clinic Institutional Review Board because it used preexisting, deidentified patient data. The study followed the Strengthening the Reporting of Observational Studies in Epidemiology (STROBE) reporting guideline.^12^

### Data Source

Medical and pharmacy claims data from the OptumLabs Data Warehouse (OLDW) were used to define the cohort. This data source includes national claims of more than 100 million privately insured individuals and Medicare Advantage enrollees in the US.^13,14^ Data include enrollment information, such as age, sex, and race/ethnicity, as well as *International Classification of Diseases, Ninth Revision, Clinical Modification* (*ICD-9-CM*) and *International Statistical Classification of Diseases, Tenth Revision, Clinical Modification* (*ICD-10-CM*) diagnosis codes; *International Classification of Diseases, Ninth Revision* (*ICD-9*) and *International Statistical Classification of Diseases and Related Health Problems, Tenth Revision* (*ICD-10*) procedure codes; *Current Procedural Terminology, Fourth Edition (CPT-4)* procedure codes; Healthcare Common Procedure Coding System procedure codes; site of service codes; and provider specialty codes (eTable 1 in the Supplement).

### Study Population

We identified a cohort of patients 18 years or older who were prescribed antithrombotic drugs (vitamin K antagonists, DOACs, and/or thienopyridine antiplatelet agents) between January 1, 2016, and December 31, 2019. We restricted the cohort to patients with no prescription in the prior 12 months and with a history of atrial fibrillation, ischemic heart disease, or venous thromboembolism. The first date of prescription fill was defined as the index prescription date. All patients had been enrolled in a health plan for 12 months before the index date. We excluded patients at risk of malignancy-associated GIB by using the presence of any cancer diagnosis code during the prior year. We also excluded patients with missing sex data and without a minimum of 12 months of follow-up after the index prescription date.

### Variables

The main outcome was the time in days to the first GIB, defined based on administrative codes^12,14^ (eTable 1 in the Supplement). The GIB events were identified by inpatient claims with 1 of these GIB administrative codes in the first or second discharge diagnosis fields, censoring at the end of enrollment (including death). All study participants were followed from the index prescription date until the end of the study period, disenrollment from insurance coverage, or the first GIB occurrence.

We considered 32 baseline (ascertained at the index date) clinical and demographic risk factors, including age, sex, race/ethnicity, condition group (atrial fibrillation, ischemic heart disease, venous thromboembolism, or combinations of these), and baseline comorbidities and medications; the complete list is provided in Table 1. For training and validation, all categorical risk factors were converted into a binary format using 1-hot encoding (ie, variables with *k* categories were transformed into *k* binary indicator variables).

**Table 1.** Characteristics of the Patients Included in Study

Characteristic	Patients, No. (%)		
	No GI bleed (n = 294 141)	GI bleed (n = 12 322)	All (N = 306 463)
Condition group			
Atrial fibrillation	46 716 (15.9)	1689 (13.7)	48 405 (15.8)
Atrial fibrillation and ischemic heart disease	68 212 (23.2)	3399 (27.6)	71 611 (23.4)
Atrial fibrillation, ischemic heart disease, and venous thromboembolism	9015 (3.1)	597 (4.8)	9612 (3.1)
Ischemic heart disease	113 136 (38.5)	4548 (36.9)	117 684 (38.4)
Ischemic heart disease and venous thromboembolism	18 423 (6.3)	938 (7.6)	19 361 (6.3)
Venous thromboembolism	34 616 (11.8)	972 (7.9)	35 588 (11.6)
Venous thromboembolism and atrial fibrillation	4023 (1.4)	179 (1.5)	4202 (1.4)
Age, mean (SD), y	68.9 (12.7)	71.2 (11.2)	69.0 (12.6)
Age group, y			
18-64	94 600 (32.2)	2871 (23.3)	97 471 (31.8)
65-74	92 352 (31.4)	4239 (34.4)	96 591 (31.5)
75-86	107 189 (36.4)	5212 (42.3)	112 401 (36.7)
Race/ethnicity			
White	185 989 (63.2)	7659 (62.2)	193 648 (63.2)
Black	31 757 (10.8)	1552 (12.6)	33 309 (10.9)
Other^a^	76 395 (26.0)	3111 (25.2)	79 506 (25.9)
Sex			
Female	133 703 (45.5)	6583 (53.4)	140 286 (45.8)
Male	160 438 (54.5)	5739 (46.6)	166 177 (54.2)
Treatment group			
Anticoagulants	166 209 (56.5)	6618 (53.7)	172 827 (56.4)
Antiplatelets	123 180 (41.9)	5519 (44.8)	128 699 (42.0)
Anticoagulants and antiplatelets	4752 (1.6)	185 (1.5)	4937 (1.6)
Baseline comorbidities			
Diabetes	122 796 (41.7)	5934 (48.2)	128 730 (42.0)
Hypertension	258 738 (88.0)	11 546 (93.7)	270 284 (88.2)
Peripheral arterial disease	45 194 (15.4)	2377 (19.3)	47 571 (15.5)
Alcoholism	18 938 (6.4)	975 (7.9)	19 913 (6.5)
Chronic kidney failure	13 615 (4.6)	922 (7.5)	14 537 (4.7)
Chronic liver disease	30 373 (10.3)	1708 (13.9)	32 081 (10.5)
Rheumatologic disease	21 033 (7.2)	1199 (9.7)	22 232 (7.3)
Carotid revascularization	13 255 (4.5)	640 (5.2)	13 895 (4.5)
*Helicobacter pylori* infection	5467 (1.9)	456 (3.7)	5923 (1.9)
History of GI bleeding	66 361 (22.6)	5217 (42.3)	71 578 (23.4)
Smoking	134 235 (45.6)	6261 (50.8)	140 496 (45.8)
Sleep apnea	37 322 (12.7)	1830 (14.9)	39 152 (12.8)
Thyroid disease	84 826 (28.8)	4049 (32.9)	88 875 (29.0)
Valvular heart disease	128 578 (43.7)	6101 (49.5)	134 679 (43.9)
Viral hepatitis	6867 (2.3)	386 (3.1)	7253 (2.4)
Percutaneous coronary intervention	21 485 (7.3)	1567 (12.7)	23 052 (7.5)
Charlson comorbidities^b^			
Cerebrovascular disease	66 909 (22.7)	3296 (26.7)	70 205 (22.9)
Dementia	17 676 (6.0)	755 (6.1)	18 431 (6.0)
Hemiplegia	12 135 (4.1)	571 (4.6)	12 706 (4.1)
AIDS	805 (0.3)	35 (0.3)	840 (0.3)
Medications			
Antiarrythmic drugs	23 640 (8.0)	1041 (8.4)	24 681 (8.1)
Antihyperlipidemic drugs	170 733 (58.0)	7592 (61.6)	178 325 (58.2)
Gastroprotective agents, proton pump inhibitors, and/or histamine 2 blockers	76 972 (26.2)	5006 (40.6)	81 978 (26.7)
Selective serotonin reuptake inhibitors	38 368 (13.0)	1962 (15.9)	40 330 (13.2)
Nonsteroidal anti-inflammatory drugs	47 964 (16.3)	1926 (15.6)	49 890 (16.3)
Antihypertensive drugs	233 458 (79.4)	10 492 (85.1)	243 950 (79.6)

Abbreviation: GI, gastrointestinal.

^a^ Other includes Asian, Hispanic, and unknown.

^b^ Defined by the Charlson Comorbidity Index.

Because we used claims data, there were no missing values among the outcomes or risk factors. Although the potential for misclassification remained because of unreported data (eg, omitted codes for patients with more complex conditions), we did not perform any sensitivity analyses to assess the potential impact of this owing to the large number of factors.

### Machine Learning Algorithms

To predict GIB using the patient characteristics described, we trained 3 machine learning algorithms for right-censored time-to-event data: regularized Cox regression (RegCox), random survival forests (RSF), and extreme gradient boosting (XGBoost). We selected these algorithms because they were commonly used for clinical models and had implementations available for time-to-event outcomes. The RegCox model is an extension of the least absolute shrinkage and selection operator (LASSO) and the Ridge regularization methods for the Cox proportional hazards regression model; RegCox penalizes models with a large number of nonzero coefficients. The RSF algorithm is an extension of the random forests algorithm for right-censored survival data.^15,16^ In RSF, multiple decision trees are trained on a random sample of observations from the study data, and the predictions are combined by using a mean value or majority vote. Unlike the traditional random forests method, in which the decision tree nodes are split by maximizing the difference in class predictions, nodes in RSF are split by maximizing differences in survival times. The XGBoost^17^ algorithm is a generalized implementation of the gradient boosting machine^18,19,20^ technique with several algorithmic enhancements to improve scalability, speed, and prediction accuracy. An important enhancement is the implementation of the LASSO and Ridge regularization, which penalizes more complex models to prevent overfitting. We implemented the Cox proportional hazards regression model partial log-likelihood as a loss function in XGBoost to analyze right-censored survival data.^21,22^ As a reference for comparing the machine learning models, we calculated the HAS-BLED risk score for each patient. Because we were using claims data, we did not have access to clinical values used in the HAS-BLED score and instead used the presence of related inpatient or outpatient claims for hypertension, abnormal kidney or liver function, stroke, history of or presence of factors associated with bleeding, labile international normalized ratio, older age (>65 years), and use of drugs or alcohol. This modified HAS-BLED score has been used previously in claims data.^23^

### Training and Validation

We divided the study sample into a development cohort (105 837 patients) and a validation cohort (200 626 patients) according to whether the index prescription date was before (development) or after (validation) January 1, 2019. The development cohort was then randomly partitioned into 10 equal subsets (folds), with random partitioning stratified by GIB status and calendar quarter. We then used the 10-fold development sample to identify the best tuning parameters (eg, the LASSO and Ridge in RegCox and XGBoost) for the RegCox, RSF, and XGBoost algorithms. Specifically, we set up a grid for each combination of parameters (chosen at random),^24^ applied the algorithm with those parameters to 9 folds of the development set, and assessed the performance of the solution on the tenth (hold-out) fold. This procedure was repeated 10 times for each parameter set in the grid (leaving out each fold in turn), and the optimal parameters were selected according to a maximum AUC over the 10 applications to the 10 hold-out folds. For each machine learning algorithm, a final model was obtained by retraining the algorithm on the entire development set using the optimal parameters selected, and the performance of this final model was assessed on the validation cohort. The workflow of the training and validation procedure and the selection of the final model are shown in eFigure 1 in the Supplement.

### Performance Measures

Using the predicted probability of GIB at 6 and 12 months from each model, we computed the sensitivity and specificity across all possible classification thresholds and constructed ROC curves. The classification threshold was a value of the predicted probability for which instances with predicted probabilities above that value were classified as GIB or as no GIB. We identified the optimal classification threshold (based on the development cohort) as the threshold value at which the distance between the point (0, 1) and the ROC curve was the minimum.^25^ We computed the accuracy (balanced), sensitivity, specificity, positive predictive value (PPV), and negative predictive value (NPV) at the optimal classification threshold, and we computed the ROC curve, AUC, cumulative gain curve, and Gini score at each time point by applying methods appropriate for censored data.^26,27^ We also developed a graphical representation of the associations among the sensitivity, PPV, and prediction density for the classification thresholds.

### Statistical Analysis

We report descriptive statistics of patient characteristics as the mean (SD) for continuous variables and as the count (percentage) for categorical variables. For each model, including the HAS-BLED model, we report the mean and 95% CI of the scalar performance and graphical performance metrics on the development cohort; these were based on the mean and SE of the 10 values. In secondary analyses, we replicated each machine learning model including HAS-BLED as a risk factor to assess whether it contributed to the model. For the best-performing machine learning model, we also report the contribution of all risk factors using a metric appropriate for the model. For this model and the HAS-BLED score, we constructed 12-month Kaplan-Meier curves using the optimal threshold to stratify the machine learning model and a score of 3 to stratify HAS-BLED.

We performed 2 sensitivity analyses to confirm the robustness of the findings. First, to assess the consequences of using time-to-event scoring in the machine learning models, we replicated all 3 approaches using classification rather than survival-time outcomes, training each model to predict 6-month and 12-month events directly. Second, to evaluate the consequences of including a heterogeneous set of conditions, we also replicated the main survival analyses using only patients with a diagnosis of atrial fibrillation.

Data analysis was performed using Stata, version 16 (StataCorp LLC), SAS, version 9.3 (SAS Institute), and R, version 3.5 (R Project for Statistical Computing). Specific R packages used were *survival*, *xgboost*, *randomForestSRC*, *ranger*, *glmnet*, and *pec*.

## Results

### Baseline Characteristics

The study sample included 306 463 patients; of these, 12 322 (4.0%) experienced a GIB event during a median follow-up of 133 days (interquartile range, 49-320 days). The mean (SD) age of the study population was 69.0 (12.6) years; 166 177 (54.2%) of the patients were men, and 193 648 (63.2%) were White. Black patients were more likely to experience GI bleeds compared with White patients and those of other races (1552 [12.6%] vs 7659 [62.2%] vs 3111 [25.2%], and female patients were more likely to experience GI bleeds than were male patients (6583 [53.4%] vs 5739 [46.6%]) (Table 1).

### Risk of GIB at 6 and 12 Months

Table 2 presents the accuracy, AUC, sensitivity, specificity, PPV, and NPV of the machine learning and HAS-BLED risk models for predicting 6- and 12-month GIB risk based on 10-fold cross-validation of the survival-based machine learning models. Consistent with the results of prior studies,^5^ the HAS-BLED score achieved an AUC of 0.61 (95% CI, 0.59-0.62) for predicting 6-month GIB risk, with similar performance for 12-month GIB risk (AUC, 0.60; 95% CI, 0.59-0.61). All 3 machine learning models had similar AUCs, although the RegCox model exhibited superior performance and significantly outperformed the HAS-BLED score in predicting 6- and 12-month GIB risk. Specifically, RegCox had an AUC of 0.68 (95% CI, 0.66-0.70) and 0.67 (95% CI, 0.65-0.69) for predicting GIB at 6 and 12 months, respectively. The RegCox and XGBoost models also showed consistently good performance in the validation cohort (RegCox: 6-month AUC, 0.67; 12-month AUC, 0.66; XGBoost: 6-month AUC, 0.67; 12-month AUC, 0.66), outperforming the HAS-BLED score (6-month AUC, 0.60; 12-month AUC, 0.59). Similar performance was observed for accuracy, sensitivity, specificity, and PPV.

**Table 2.** Model Evaluation and Validation

Model	Month	AUC (95% CI)	Sensitivity (95% CI)	Specificity (95% CI)	PPV (95% CI)	NPV (95% CI)	Balance accuracy (95% CI)^a^	Cutoff^b^
**XGBoost**								
Development cohort	6	0.68 (0.66-0.69)	0.45 (0.03-0.97)	0.71 (0.11-0.99)	0.06 (0.03-0.11)	0.98 (0.97-0.99)	0.58 (0.51-0.64)	0.02 (0.02, 0.02)
	12	0.67 (0.65-0.68)	0.65 (0.21-0.97)	0.52 (0.10-0.90)	0.06 (0.04-0.08)	0.98 (0.97-0.99)	0.59 (0.53-0.61)	0.03 (0.03, 0.03)
Validation cohort^c^	6	0.67	0.26	0.9	0.05	0.98	0.58	0.02
	12	0.66	0.59	0.66	0.04	0.99	0.62	0.03
**RSF**								
Development cohort	6	0.66 (0.64-0.69)	0.58 (0.42-0.72)	0.65 (0.51-0.80)	0.05 (0.04-0.06)	0.98 (0.98-0.98)	0.62 (0.61-0.64)	0.04 (0.03, 0.04)
	12	0.65 (0.62-0.67)	0.62 (0.55-0.67)	0.60 (0.54-0.64)	0.06 (0.05-0.06)	0.98 (0.97-0.98)	0.61 (0.59-0.62)	0.06 (0.05, 0.07)
Validation cohort^c^	6	0.62	0.53	0.65	0.03	0.99	0.59	0.04
	12	0.60	0.58	0.58	0.03	0.98	0.58	0.06
**RegCox**								
Development cohort	6	0.68 (0.66-0.70)	0.67 (0.64-0.70)	0.59 (0.58-0.59)	0.04 (0.04-0.05)	0.98 (0.98-0.99)	0.63 (0.62-0.64)	0.03 (0.03, 0.03)
	12	0.67 (0.65-0.69)	0.67 (0.65-0.70)	0.56 (0.55-0.57)	0.06 (0.05-0.06)	0.98 (0.97-0.98)	0.62 (0.61-0.63)	0.05 (0.05, 0.05)
Validation cohort^c^	6	0.67	0.69	0.57	0.03	0.99	0.63	0.03
	12	0.66	0.7	0.54	0.03	0.99	0.62	0.05
**HAS-BLED**								
Development cohort	6	0.61 (0.59-0.62)	0.54 (0.51-0.56)	0.62 (0.62-0.63)	0.03 (0.03-0.04)	0.98 (0.98-0.99)	0.58 (0.56-0.59)	3
	12	0.60 (0.59-0.61)	0.53 (0.51-0.54)	0.62 (0.62-0.63)	0.05 (0.04-0.05)	0.98 (0.97-0.98)	0.57 (0.56-0.58)	3
Validation cohort^c^	6	0.60	0.57	0.58	0.02	0.99	0.56	3
	12	0.59	0.56	0.58	0.03	0.99	0.56	3

Abbreviations: AUC, area under the receiving operator characteristic curve; HAS-BLED, hypertension, abnormal kidney or liver function, stroke, history of and factors associated with presence of bleeding, labile international normalized ratio, older age (>65 years), use of drugs or alcohol concomitantly; NPV, negative predictive value; PPV, positive predictive value; RegCox, regularized Cox proportional hazards regression; RSF, random survival forests; XGBoost, extreme gradient boosting.

^a^ The balanced accuracy is the arithmetic mean of the sensitivity and specificity design to better judge the performance of a classifier (compared with the simple accuracy, which is the percentage of the correctly classified metric), especially in a setting in which the classes are highly imbalanced.

^b^ The cutoff is the classification threshold that minimizes the distance between the receiver operating characteristic curve and the upper left corner of the graph or the point (0, 1).

^c^ Because there was only 1 validation data set, results for the validation cohort are expressed as point estimates without 95% CIs.

**Figure 1.**
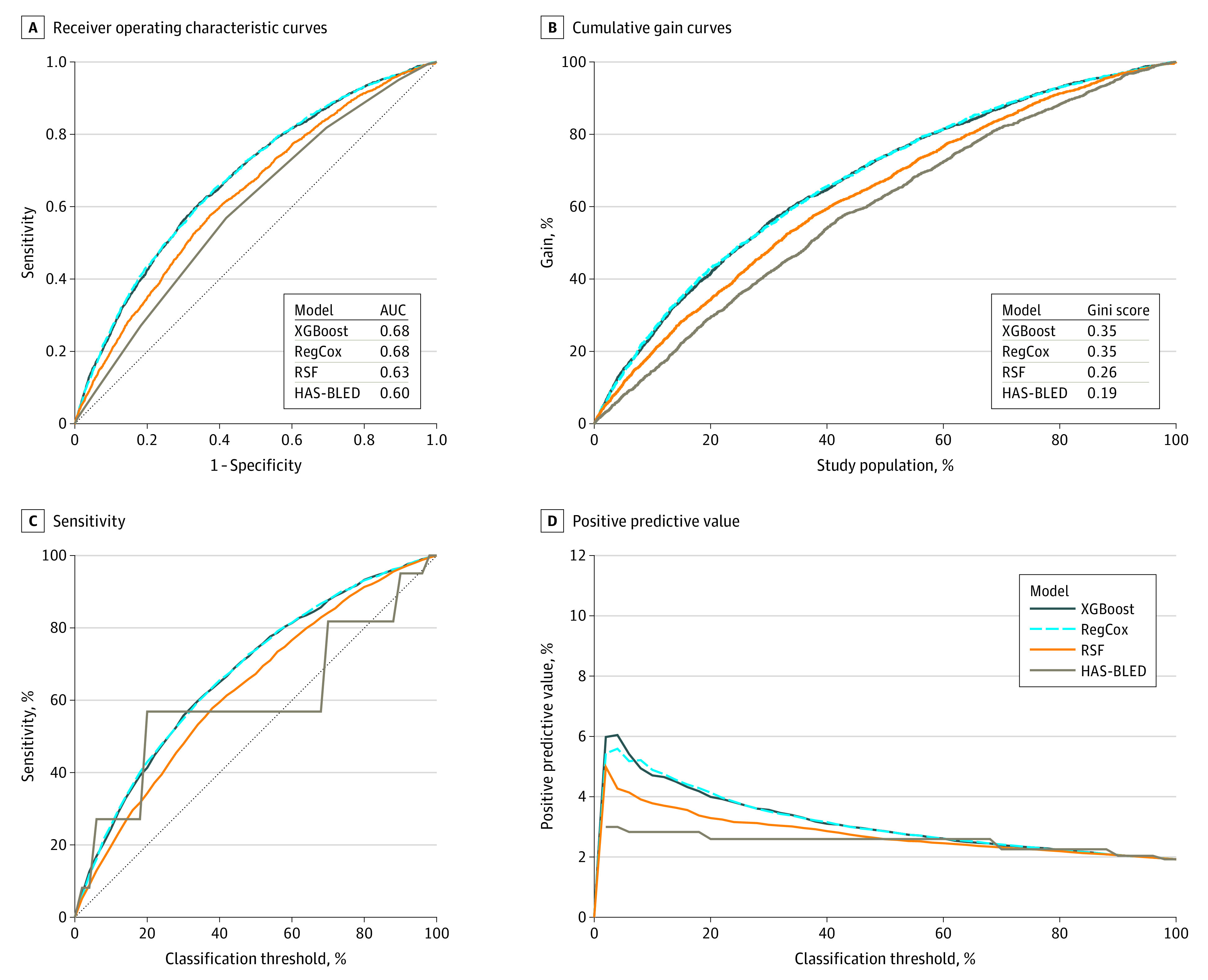
Performance Plots for Predicting Gastrointestinal Bleeding at 12 Months in the Development Cohort The Gini score was computed by dividing the area between the gain curve and the random classifier (dotted diagonal line) by the area between the perfect classifier and the random classifier. AUC indicates area under the ROC curve; HAS-BLED, hypertension, abnormal kidney or liver function, stroke, history of and factors associated with presence of bleeding, labile international normalized ratio, older age (>65 years), use of drugs or alcohol concomitantly; RegCox, regularized Cox proportional hazards regression; RSF, random survival forests; XGBoost, extreme gradient boosting.

Figure 1 shows the ROC, cumulative gain, sensitivity, and PPV curves for the machine learning and HAS-BLED models in predicting 12-month GIB risk in the development data set (with mean values determined over the 10-fold cross-validations). The ROC curves reflect higher AUCs for all 3 machine learning models compared with HAS-BLED. The gain curve shows that if the top 10% of the entire population were selected as high-risk for GIB at 12 months based on the RegCox predictions, the sample would contain approximately 25% of actual patients at high-risk for GB; in comparison, the HAS-BLED predictions would contain only 15% of actual high-risk cases. The sensitivity and PPV curves of the machine learning models further confirmed this, being substantially higher than the HAS-BLED curves across most of the classification thresholds. The secondary analyses including HAS-BLED as a factor in the machine learning models gave very similar results, with identical development AUCs at 6 months for the 3 models and slightly lower 12-month AUCs.

The results of the sensitivity analyses based on the classification models were similar, with validation AUCs for the machine learning methods ranging from 0.66 to 0.69, whereas the HAS-BLED model had 6- and 12-month validation AUCs of 0.60 and 0.59, respectively (eTable 2 in the Supplement). The results using only the patients with atrial fibrillation were also similar, with the machine learning AUCs ranging from 0.60 to 0.67 and the HAS-BLED model having 6- and 12-month validation AUCs of 0.60 and 0.59, respectively (eTable 3 in the Supplement).

**Figure 2.**
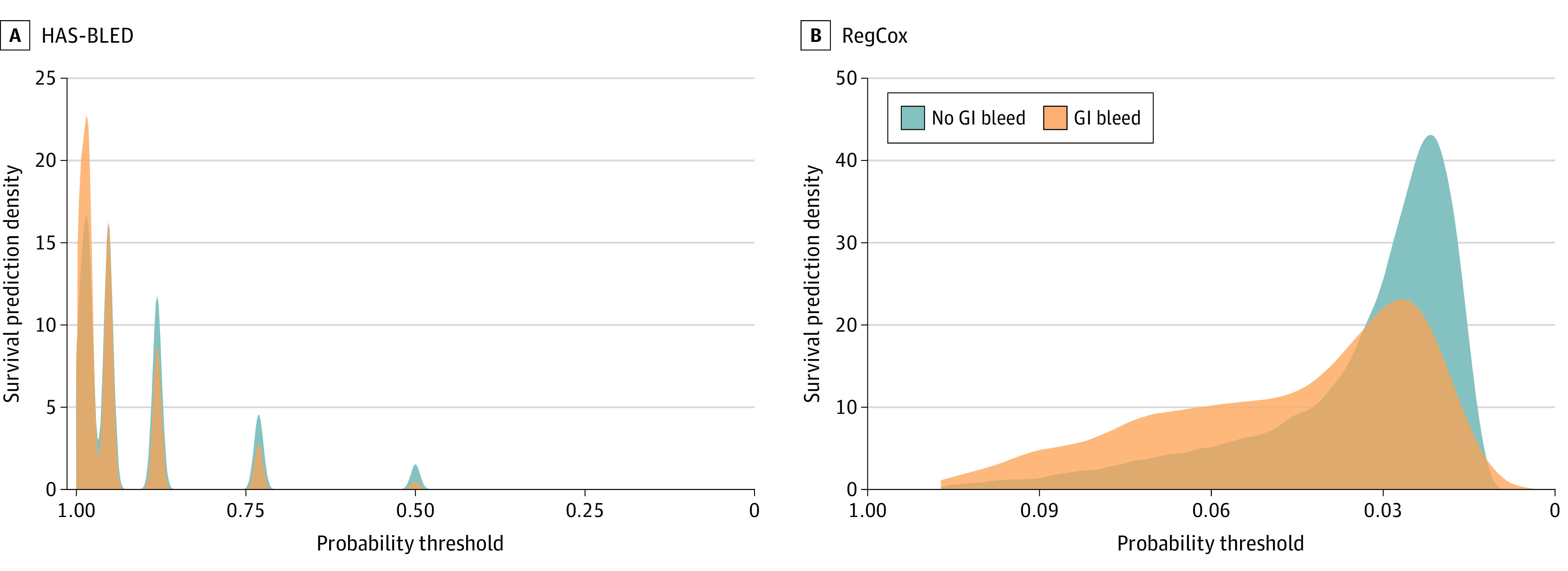
Survival Prediction Density by Gastrointestinal (GI) Bleed Status at 12 Months for HAS-BLED and Regularized Cox Proportional Hazards Regression (RegCox) Models HAS-BLED indicates hypertension, abnormal kidney or liver function, stroke, history of and factors associated with presence of bleeding, labile international normalized ratio, older age (>65 years), use of drugs or alcohol concomitantly.

Figure 2 shows the prediction density of the HAS-BLED and RegCox models to better compare the best- and worst-performing models. The predicted class probability density plot for the HAS-BLED score showed a significant overlap between the classes, indicating an inability to discriminate the classes. In contrast, the plots for RegCox showed less overlap between the class distributions.

### Importance Scores

**Figure 3.**
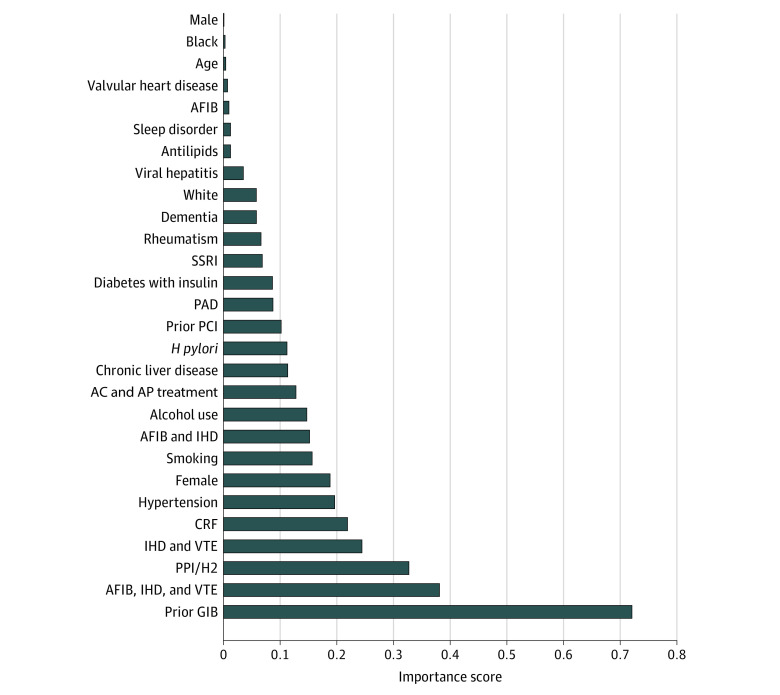
Importance Scores for Factors Included in the Regularized Cox Proportional Hazards Regression (RegCox) Machine Learning Model The importance scores (β coefficients) from the 12-month RegCox model for all factors are shown. AC indicates anticoagulants; AFIB, atrial fibrillation; AP, antiplatelets; CRF, chronic renal failure; GIB, gastrointestinal bleeding; H2, histamine 2 blocker; IHD, ischemic heart disease; PAD, peripheral arterial disease; PCI, percutaneous coronary intervention; PPI, proton pump inhibitor; SSRI, selective serotonin reuptake inhibitor; VTE, venous thromboembolism.

Given the overall better performance of RegCox, we graphed the importance scores (β coefficients) from the 12-month model for all factors to assess face validity (Figure 3). The variables with the highest importance scores in the RegCox model were prior GI bleed (0.72); atrial fibrillation, ischemic heart disease, and venous thromboembolism combined (0.38); and use of gastroprotective agents (0.32). Results were similar for the 6-month model. The Kaplan-Meier curves for the RegCox and HAS-BLED models are shown in eFigures 2 and 3 in the Supplement.

## Discussion

In this study of more than 300 000 patients receiving treatment with antithrombotic agents, we found that machine learning techniques modestly outperformed the HAS-BLED score in discriminating patients at risk of GIB after prescription of the antithrombotic agent at both 6 and 12 months. Of the 3 machine learning approaches, the RegCox model had marginally better discrimination than the others. Of more importance, this RegCox model for predicting GIB risk at 6 and 12 months performed substantially better in this population than the current standard risk model, HAS-BLED.

Using claims data from the OLDW, we were able to construct a larger cohort with longer follow-up than, to our knowledge, had been used before in model development, and the cohort included patients who received treatment with a range of antithrombotic agents, thus improving the generalizability and clinical relevance of the results. We constructed prediction models using 3 machine learning approaches. By comparing these models with each other as well as with the best model from the literature, we were able to evaluate the incremental advantage, if any, of applying machine learning techniques for risk stratification. By comparing machine learning models with the HAS-BLED model, we were also able to identify whether any of these approaches improved on the established risk model.

There has been increasing interest in better understanding the impact of machine learning approaches in the context of clinical prediction models. Christodoulou et al^28^ undertook a systematic review of comparisons of machine learning methods with logistic regression and found no evidence that machine learning models performed better than logistic regression; the mean difference in AUCs between machine learning and logistic regression was 0.00 (95% CI, −0.18 to 0.18). A systematic review by Shung et al^29^ of machine learning approaches to predict outcomes in patients with existing GIB included a separate set of studies than those reviewed by Christodoulou et al^28^ and found that artificial neural networks generally outperformed all other methods, although this conclusion was based on a single study^30^ of artificial neural networks that did not use a comparator approach. A later, more limited comparison^31^ of machine learning approaches for coronary heart disease found that neural nets and vector machines performed better than random forests, but the performance of both depended on the software implementation. The current study’s findings add to these systematic comparisons of machine learning methods using a large data set with a time-to-event outcome.

The optimal model in this study, RegCox, performed marginally better than not only other machine learning approaches but also a version of HAS-BLED, the most commonly used existing model. Given that we modified the HAS-BLED model to use claims-based risk factors, this finding should not be interpreted as a direct comparison with the established clinical model, but the finding does suggest that machine learning approaches can improve on standard approaches if the same data are used. Furthermore, other machine learning approaches might produce better-performing prediction models. For example, both extreme-learning machine Cox proportional hazards regression models^32^ and super-learning survival models^33^ have been found to perform better than any of the individual methods tested in this study. We chose the 3 machine learning approaches for this study because of their appropriateness for the data and their somewhat widespread use for constructing risk prediction models. However, the finding that the 3 approaches performed better than the standard model (HAS-BLED) for predicting GIB might be further supported by the use of alternative super- or extreme-learning approaches. Of more importance, we found little difference in the performance profiles of the 3 machine learning approaches, suggesting that there may be little gain in comparing models for future risk profiling.

All of the approaches used in this study, including the HAS-BLED, had low PPVs and high NPVs, indicating that all of the models are better at identifying patients who will not experience a GIB than at identifying those who will. This suggests that using any of these models for clinical decision-making will be most appropriate for identifying patients at low risk. The moderate AUCs of these models indicate that they should be considered as supplementary to other input for clinical decision-making because they all had a limited ability to discriminate. This study’s findings should be viewed primarily as informing the development of better risk models for GIB.

Of greater importance is the relative performance of the RegCox model, which this study found to be the best risk model for GIB. The current study included more than 300 000 patients, making it, to our knowledge, 1 of the largest analyses of GIB risk. Each fold of the 10-fold data set included nearly 20 000 patients, more than was included overall in most prior studies of GIB. With use of this cohort, the RegCox model performed only marginally better than the modified HAS-BLED risk prediction score at predicting the 6- and 12-month risk of GIB, with AUCs at 6 and 12 months of 0.67 and 0.66, respectively, compared with AUCs of 0.60 and 0.59 for the HAS-BLED model. All of the AUCs were less than 0.70, a conventionally used threshold for acceptable performance^34^; however, these values were consistent with AUCs for predicting the same outcome using other cohorts, data, and methods as well as HAS-BLED.^35,36^ A key unknown factor is whether additional data could further improve the performance of either statistical or machine learning models. For example, imaging data from endoscopies or self-reported data about patient behaviors might increase the ability to predict the risk of GIB in patients being considered for antithrombotic therapies. Existing data sets are not able to address this question but will be important in future studies of combinations of novel data and novel analytics to improve the delivery of health care.

Although we were able to construct a GIB risk prediction model with improved sensitivity and specificity using machine learning methods, the choice of method was not critical to the model’s performance. The machine learning models showed improvement over the existing HAS-BLED model and could serve as the basis for a clinical tool for assessing GIB risk.

### Limitations

This study has limitations. The OLDW is one of the largest and most representative claims databases available, but it does not include patients who are uninsured or, more important, who are insured by Medicare. By excluding this large cohort of older patients, this study’s findings may be less easily generalized to older patients. The OLDW includes a large number of patients who are privately insured or covered by Medicare Advantage, and it is unlikely that the exclusion of publicly insured patients biased the comparisons among machine learning approaches. Also, the inclusion of other factors such as laboratory data not only might improve the performance of all machine learning approaches but might do so differentially. This does not undermine the current findings but rather points toward future areas of research.

## Conclusions

In this cross-sectional study, the machine learning models examined showed similar performance in identifying patients at high risk for GIB after being prescribed antithrombotic agents. Two models (RegCox and XGBoost) performed modestly better than the HAS-BLED score. A prospective evaluation of the RegCox model compared with HAS-BLED may provide a better understanding of the clinical impact of improved performance. Furthermore, the findings suggest that developers of risk prediction tools should consider machine learning algorithms, but 1 machine learning technique might not be clearly superior to another. Future prospective studies appear to be needed to better understand the extent of improvement in predictive performance that can also improve clinical outcomes.

## Notes

Supplement. **eFigure 1.** Workflow of training, evaluation, and validation of ML models **eFigure 2.** Kaplan-Meier 12-month survival curves for the RegCox model **eFigure 3.** Kaplan-Meier 12-month survival curves for the HAS-BLED model **eTable 1.** *ICD-9* and *ICD-10* diagnostic codes used **eTable 2.** Performance results for classification models **eTable 3.** Performance of time-to-event models, developed and validated using only patients with AF

## References

[1] Chen L. Overview of clinical prediction models. Ann Transl Med. 2020;8(4):71. doi:10.21037/atm.2019.11.121 32175364 PMC7049012

[2] Koskinas KC, Räber L, Zanchin T, . Clinical impact of gastrointestinal bleeding in patients undergoing percutaneous coronary interventions. Circ Cardiovasc Interv. 2015;8(5):e002053. doi:10.1161/CIRCINTERVENTIONS.114.002053 25910501

[3] Sørensen R, Hansen ML, Abildstrom SZ, . Risk of bleeding in patients with acute myocardial infarction treated with different combinations of aspirin, clopidogrel, and vitamin K antagonists in Denmark: a retrospective analysis of nationwide registry data. Lancet. 2009;374(9706):1967-1974. doi:10.1016/S0140-6736(09)61751-7 20006130

[4] Roldán V, Marín F, Fernández H, . Predictive value of the HAS-BLED and ATRIA bleeding scores for the risk of serious bleeding in a “real-world” population with atrial fibrillation receiving anticoagulant therapy. Chest. 2013;143(1):179-184. doi:10.1378/chest.12-0608 22722228

[5] Pisters R, Lane DA, Nieuwlaat R, de Vos CB, Crijns HJ, Lip GY. A novel user-friendly score (HAS-BLED) to assess 1-year risk of major bleeding in patients with atrial fibrillation: the Euro Heart Survey. Chest. 2010;138(5):1093-1100. doi:10.1378/chest.10-0134 20299623

[6] Senoo K, Proietti M, Lane DA, Lip GY. Evaluation of the HAS-BLED, ATRIA, and ORBIT bleeding risk scores in patients with atrial fibrillation taking warfarin. Am J Med. 2016;129(6):600-607. doi:10.1016/j.amjmed.2015.10.001 26482233

[7] Apostolakis S, Lane DA, Guo Y, Buller H, Lip GY. Performance of the HEMORR(2)HAGES, ATRIA, and HAS-BLED bleeding risk-prediction scores in patients with atrial fibrillation undergoing anticoagulation: the AMADEUS (evaluating the use of SR34006 compared to warfarin or acenocoumarol in patients with atrial fibrillation) study. J Am Coll Cardiol. 2012;60(9):861-867. doi:10.1016/j.jacc.2012.06.019 22858389

[8] Qiu J, Grine K. Assessing bleeding risk in patients taking anticoagulants. Am Fam Physician. 2017;96(7):465-466. 29094904

[9] Faye A, Hung K, Cheng K, . 626 HAS-BLED scores underestimate gastrointestinal bleeding risk among those with H. pylori. Am J Gastroenterol. 2019;114:S364. doi:10.14309/01.ajg.0000592040.17612.c1

[10] Bahat G, İlhan B, Karan M. HAS-BLED score: limitations due to underestimation of bleeding risk in the elderly. Nobel Medicus. 2015;11:101-102. Accessed May 4, 2021. https://www.nobelmedicus.com/Content/1/32/101-102.pdf

[11] Abraham NS, Noseworthy PA, Inselman J, . Risk of gastrointestinal bleeding increases with combinations of antithrombotic agents and patient age. Clin Gastroenterol Hepatol. 2020;18(2):337-346.e19. doi:10.1016/j.cgh.2019.05.017 31108228 PMC7386161

[12] Vandenbroucke JP, von Elm E, Altman DG, ; STROBE initiative. Strengthening the Reporting of Observational Studies in Epidemiology (STROBE): explanation and elaboration. Ann Intern Med. 2007;147(8):W163-194. doi:10.7326/0003-4819-147-8-200710160-00010-w1 17938389

[13] Abraham NS, Noseworthy PA, Yao X, Sangaralingham LR, Shah ND. Gastrointestinal safety of direct oral anticoagulants: a large population-based study. Gastroenterology. 2017;152(5):1014-1022.e1. doi:10.1053/j.gastro.2016.12.018 28043907

[14] Abraham NS, Singh S, Alexander GC, . Comparative risk of gastrointestinal bleeding with dabigatran, rivaroxaban, and warfarin: population based cohort study. BMJ. 2015;350:h1857. doi:10.1136/bmj.h1857 25910928 PMC4413863

[15] Ishwaran H, Kogalur UB, Blackstone EH, Lauer MS. Random survival forests. Ann Appl Stat. 2008;2(3):841-860. doi:10.1214/08-AOAS169

[16] Breiman L. Random forests. Mach Learn. 2001;45(1):5-32. doi:10.1023/A:1010933404324

[17] Chen T, Guestrin C. XGBoost: a scalable tree boosting system. In: *Proceedings of the 22nd ACM SIGKDD International Conference on Knowledge Discovery and Data Mining; San Francisco, California, USA*. Association for Computing Machinery; 2016:785-794.

[18] Freund Y, Schapire R, Abe N. A short introduction to boosting. Journal of JSAI. 1999;14(1612):771-780. Accessed May 4, 2021. https://cseweb.ucsd.edu/~yfreund/papers/IntroToBoosting.pdf

[19] Freund Y, Schapire RE. Experiments with a new boosting algorithm. In: Machine Learning: Proceedings of the Thirteenth International Conference (ICML '96). Morgan Kaufman; 1996:148-156.

[20] Friedman JH. Greedy function approximation: a gradient boosting machine. Ann Statist. 2001;29(5):1189-232. doi:10.1214/aos/1013203451

[21] Ridgeway G. Generalized boosted models: a guide to the gbm package. Compute (Greensboro). 2007;1(4):1-12. Accessed May 4, 2021. https://cran.r-project.org/web/packages/gbm/vignettes/gbm.pdf

[22] Ridgeway G. The state of boosting. Comput Sci Stat. 1991;31:172-181. Accessed May 4, 2021. http://www.ressources-actuarielles.net/EXT/ISFA/1226.nsf/0/a29acbd26d902d6fc125822a0031c09b/$FILE/boosting.pdf

[23] Yao X, Gersh BJ, Sangaralingham LR, . Comparison of the CHA_2_DS_2_-VASc, CHADS_2_, HAS-BLED, ORBIT, and ATRIA risk scores in predicting non-vitamin K antagonist oral anticoagulants-associated bleeding in patients with atrial fibrillation. Am J Cardiol. 2017;120(9):1549-1556. doi:10.1016/j.amjcard.2017.07.051 28844514

[24] Bergstra J, Bengio Y. Random search for hyper-parameter optimization. J Mach Learn Res. 2012;13:281-305. Accessed May 4, 2021. https://www.jmlr.org/papers/volume13/bergstra12a/bergstra12a.pdf

[25] Song B, Zhang G, Zhu W, Liang Z. ROC operating point selection for classification of imbalanced data with application to computer-aided polyp detection in CT colonography. Int J Comput Assist Radiol Surg. 2014;9(1):79-89. doi:10.1007/s11548-013-0913-8 23797823 PMC3835757

[26] Greene HJ, Milne GR. Assessing model performance: the Gini statistic and its standard error. J Database Marketing Customer Strategy Management. 2010;17(1):36-48. doi:10.1057/dbm.2010.2

[27] Kamarudin AN, Cox T, Kolamunnage-Dona R. Time-dependent ROC curve analysis in medical research: current methods and applications. BMC Med Res Methodol. 2017;17(1):53. doi:10.1186/s12874-017-0332-6 28388943 PMC5384160

[28] Christodoulou E, Ma J, Collins GS, Steyerberg EW, Verbakel JY, Van Calster B. A systematic review shows no performance benefit of machine learning over logistic regression for clinical prediction models. J Clin Epidemiol. 2019;110:12-22. doi:10.1016/j.jclinepi.2019.02.004 30763612

[29] Shung D, Simonov M, Gentry M, Au B, Laine L. Machine learning to predict outcomes in patients with acute gastrointestinal bleeding: a systematic review. Dig Dis Sci. 2019;64(8):2078-2087. doi:10.1007/s10620-019-05645-z 31055722

[30] Ali A, Swingland J, Choi CH, . OC-143 artificial neural network for the risk stratification of acute upper gastrointestinal bleeding: multicentre comparative analysis vs the Glasgow Blatchford and rockall scores. Gut. 2012;61(suppl 2):A62. doi:10.1136/gutjnl-2012-302514a.143

[31] Beunza JJ, Puertas E, García-Ovejero E, . Comparison of machine learning algorithms for clinical event prediction (risk of coronary heart disease). J Biomed Inform. 2019;97:103257. doi:10.1016/j.jbi.2019.103257 31374261

[32] Wang H, Li G. Extreme learning machine Cox model for high-dimensional survival analysis. Stat Med. 2019;38(12):2139-2156. doi:10.1002/sim.8090 30632193 PMC6498851

[33] Golmakani MK, Polley EC. Super learner for survival data prediction. Int J Biostat. Published online February 22, 2020. doi:10.1515/ijb-2019-0065 32097120

[34] Hosmer DW, Lemeshow S. *Applied Logistic Regression*. 2nd ed. John Wiley & Sons; 2000:143-202.

[35] Donzé J, Rodondi N, Waeber G, Monney P, Cornuz J, Aujesky D. Scores to predict major bleeding risk during oral anticoagulation therapy: a prospective validation study. Am J Med. 2012;125(11):1095-1102. doi:10.1016/j.amjmed.2012.04.005 22939362

[36] O’Brien EC, Simon DN, Thomas LE, . The ORBIT bleeding score: a simple bedside score to assess bleeding risk in atrial fibrillation. Eur Heart J. 2015;36(46):3258-3264. doi:10.1093/eurheartj/ehv476 26424865 PMC4670965

