# Supplementary material for: Comparative Effectiveness of Machine Learning Approaches for Predicting Gastrointestinal Bleeds in Patients Receiving Antithrombotic Treatment
Source: JAMA Netw Open. 2021 May 21;4(5):e2110703. doi: 10.1001/jamanetworkopen.2021.10703 (PMC8140376; doi:10.1001/jamanetworkopen.2021.10703)
Supplement: Supplement. — eFigure 1. Workflow of training, evaluation, and validation of ML models eFigure 2. Kaplan-Meier 12-month survival curves for the RegCox model eFigure 3. Kaplan-Meier 12-month survival curves for the HAS-BLED model eTable 1. ICD-9 and ICD-10 diagnostic codes used eTable 2. Performance results for classification models eTable 3. Performance of time-to-event models, developed and validated using only patients with AF [file jamanetwopen-e2110703-s001.pdf]

Supplementary Online Content

Herrin J, Abraham NS, Yao X, et al. Comparative effectiveness of machine learning approaches for predicting gastrointestinal bleeds in patients receiving antithrombotic treatment. *JAMA Netw Open*. 2021;4(5):e2110703. doi:10.1001/jamanetworkopen.2021.10703

eFigure 1. Workflow of training, evaluation, and validation of ML models

eFigure 2. Kaplan-Meier 12-month survival curves for the RegCox model

eFigure 3. Kaplan-Meier 12-month survival curves for the HAS-BLED model

eTable 1. *ICD-9* and *ICD-10* diagnostic codes used

eTable 2. Performance results for classification models

eTable 3. Performance of time-to-event models, developed and validated using only patients with AF

This supplementary material has been provided by the authors to give readers additional information about their work.

© 2021 Herrin J et al. *JAMA Network Open*.

eFigure 1.

Workflow of training, evaluation and validation of ML models. The development data were randomly partitioned into 10 equal and independent parts (folds). For each of the 10 folds, we trained the models on the other 9 folds and evaluated on the remaining fold. For each 9-fold training set, we selected optimal tuning parameters for each model and evaluated performance on the hold-out fold. The parameters which gave optimal performance over all 10 evaluations were used to re-train the model on the complete (10-fold) development data. This final model was then evaluated on the validation data. The same process was replicated for each ML approach.

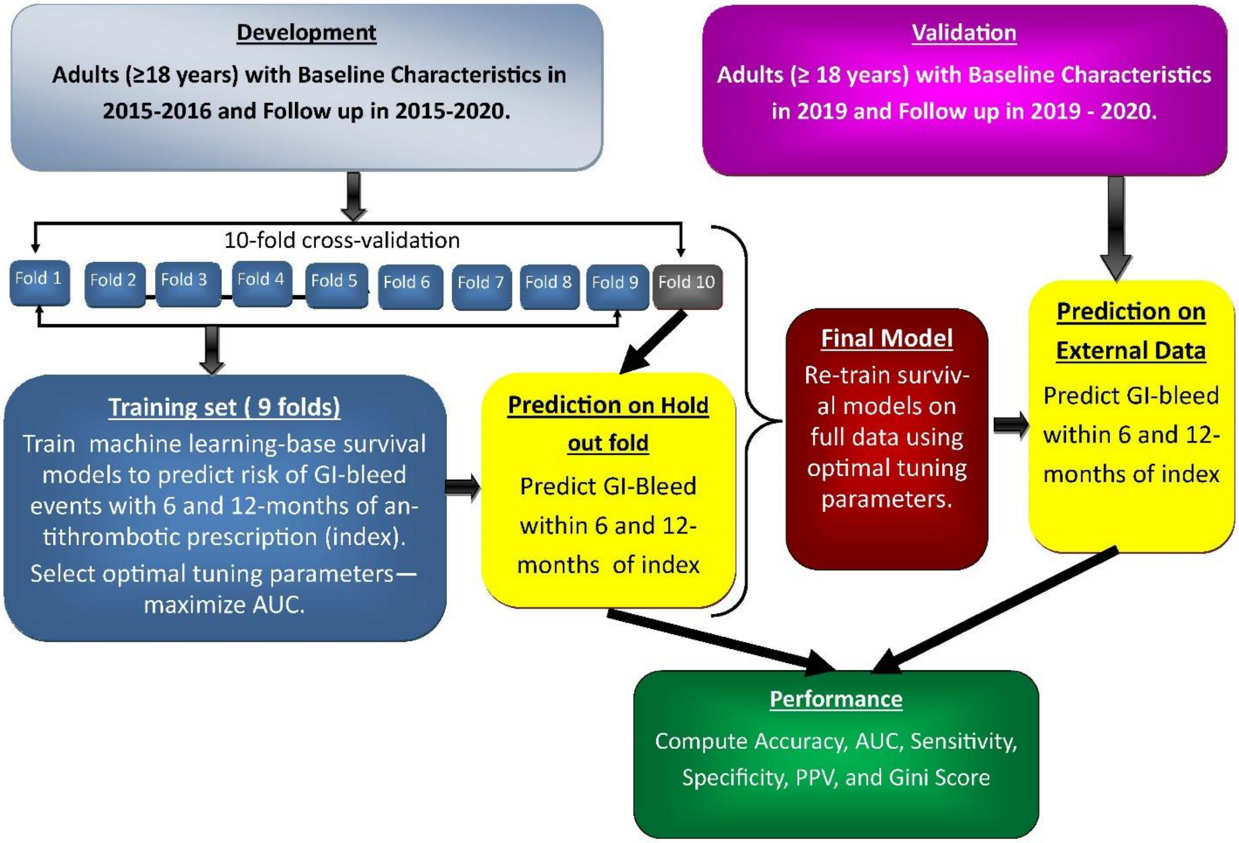

© 2021 Herrin J et al. *JAMA Network Open*.

eFigure 2.

Kaplan-Meier 12-month survival curves for the RegCox model. The RegCox curves are stratified on the optimal discrimination threshold for the RegCox model.

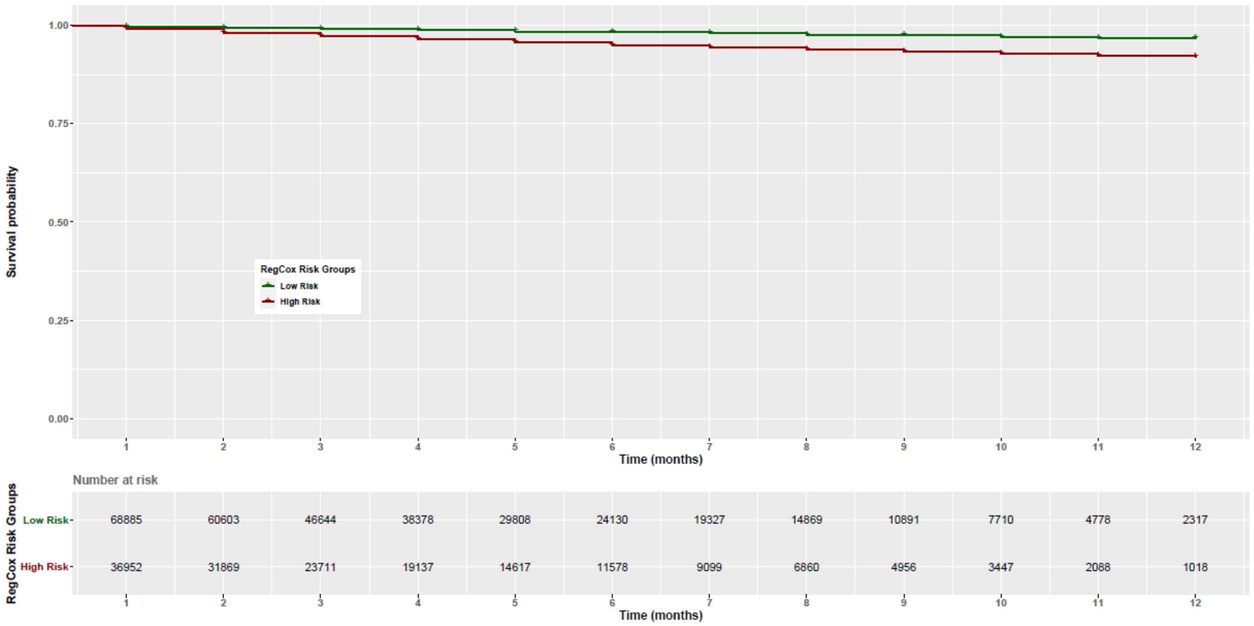

© 2021 Herrin J et al. *JAMA Network Open*.

eFigure 3. Kaplan-Meier 12-month survival curves for the HAS-BLED model. The HAS-BLED curves are stratified on score <=3 vs > 3.

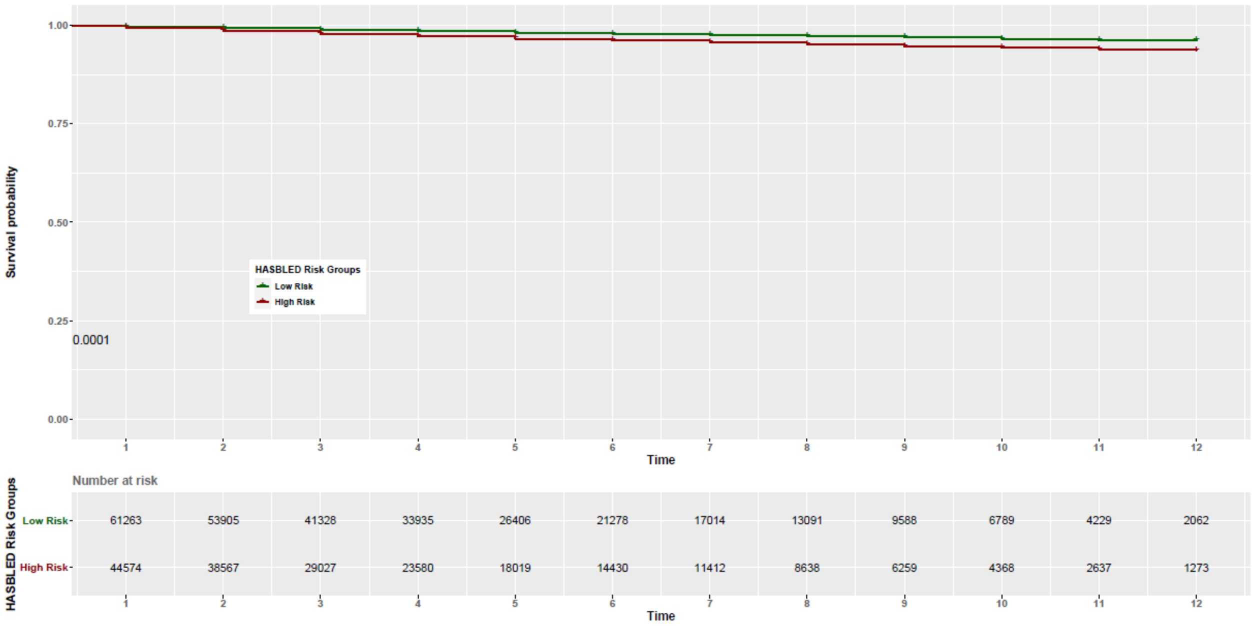

© 2021 Herrin J et al. *JAMA Network Open*.

eTable 1. *ICD-9* and *ICD-10* Diagnostic Codes Used

These codes were used to identify the outcome, GI Bleed.

Upper GI Bleed			Lower GI Bleed		
**Code**	**Code Version**	**Code Description**	**Code**	**Code Version**	**Code Description**
456.2	9	ESOPHAGEAL VARICES DISEASES CLASSIFIED ELSEWHERE	562.02	9	DIVERTICULOSIS OF SMALL INTESTINE WITH HEMORRHAGE
530.7	9	GASTROESOPHAGEAL LACERATION-HEMORRHAGE SYNDROME	562.03	9	DIVERTICULITIS OF SMALL INTESTINE WITH HEMORRHAGE
530.82	9	ESOPHAGEAL HEMORRHAGE	562.12	9	DIVERTICULOSIS OF COLON WITH HEMORRHAGE
531.0	9	ACUTE GASTRIC ULCER WITH HEMORRHAGE	562.13	9	DIVERTICULITIS OF COLON WITH HEMORRHAGE
531.00	9	ACUTE GASTRIC ULCER WITH HEMORRHAGE WITHOUT MENTION OBSTRUCTION	568.81	9	HEMOPERITONEUM
531.01	9	ACUTE GASTRIC ULCER WITH HEMORRHAGE AND OBSTRUCTION	569.3	9	HEMORRHAGE OF RECTUM AND ANUS
531.1	9	ACUTE GASTRIC ULCER WITH PERFORATION	569.85	9	ANGIODYSPLASIA OF INTESTINE WITH HEMORRHAGE
531.10	9	ACUTE GASTRIC ULCER WITH PERFORATION WITHOUT MENTION OBSTRUCTION	K55.21	10	ANGIODYSPLASIA OF COLON WITH HEMORRHAGE
531.11	9	ACUTE GASTRIC ULCER WITH PERFORATION AND OBSTRUCTION	K57.01	10	DIVERTICULITIS SMALL INTESTINE WITH PERFORATION AND ABSCESS WITH BLEED
531.2	9	ACUTE GASTRIC ULCER WITH HEMORRHAGE AND PERFORATION	K57.11	10	DIVERTICULOSIS SMALL INTESTINE WITHOUT PERFORATION/ ABSCESS WITH BLEED
531.20	9	ACUTE GASTRIC ULCER WITH HEMORRHAGE PERFORATION WITHOUT MENTION OBSTRUCTION	K57.13	10	DIVERTICULITIS SMALL INTESTINE WITHOUT PERFORATION/ ABSCESS WITH BLEED
531.21	9	ACUTE GASTRIC ULCER WITH HEMORRHAGE PERFORATION AND OBSTRUCTION	K57.21	10	DIVERTICULITIS LARGE INTESTINE WITH PERFORATION AND ABSCESS WITH BLEED
531.3	9	ACUTE GASTRIC ULCER WITHOUT MENTION HEMORRHAGE PERFORATION	K57.31	10	DIVERTICULOSIS LARGE INTESTINE WITHOUT PERFORATION/ ABSCESS WITH BLEED
531.30	9	ACUTE GASTRIC ULCER WITHOUT MENTION HEMORRHAGE PERFORATION/OBSTRUCTION	K57.33	10	DIVERTICULITIS LARGE INTESTINE WITHOUT PERFORATION/ ABSCESS WITH BLEED
531.31	9	ACUTE GASTRIC ULCER WITHOUT MENTION HEMORRHAGE/PERFORATION WITH OBSTRUCTION	K57.41	10	DIVERTICULITIS SMALL AND LARGE INTESTINE WITH PERFORATION AND ABSCESS WITH BLEED
531.4	9	CHRONIC/UNSPECIFIED GASTRIC ULCER WITH HEMORRHAGE	K57.51	10	DIVERTICULOSIS SMALL AND LARGE INTESTINE WITHOUT PERFORATION/ ABSCESS WITH BLEED
531.40	9	CHRONIC/UNSPECIFIED GASTRIC ULCER WITH HEMORRHAGE WITHOUT MENTION OBSTRUCTION	K57.53	10	DIVERTICULITIS SMALL AND LARGE INTESTINE WITHOUT PERFORATION/ ABSCESS WITH BLEED
531.41	9	CHRONIC/UNSPECIFIED GASTRIC ULCER WITH HEMORRHAGE AND OBSTRUCTION	K57.91	10	DIVERTICULOSIS PART UNSPECIFIED WITHOUT PERFORATION OR ABSCESS WITH BLEED
531.5	9	CHRONIC/UNSPECIFIED GASTRIC ULCER WITH PERFORATION	K57.93	10	DIVERTICULITIS PART UNSPECIFIED WITHOUT PERFORATION OR ABSCESS WITH BLEED
531.50	9	CHRONIC/UNSPECIFIED GASTRIC ULCER WITH PERFORATION WITHOUT MENTION OBSTRUCTION	K62.5	10	HEMORRHAGE OF ANUS AND RECTUM
531.51	9	CHRONIC/UNSPECIFIED GASTRIC ULCER WITH PERFORATION AND OBSTRUCTION	K66.1	10	HEMOPERITONEUM
531.6	9	CHRONIC/UNSPECIFIED GASTRIC ULCER WITH HEMORRHAGE AND PERFORATION			
531.60	9	CHRONIC/UNSPECIFIED GASTRIC ULCER WITH HEMORRHAGE AND PERFORATION WITHOUT OBSTRUCTION			
531.61	9	CHRONIC/UNSPECIFIED GASTRIC ULCER WITH HEMORRHAGE PERFORATION AND OBSTRUCTION			
532.0	9	ACUTE DUODENAL ULCER WITH HEMORRHAGE			
532.00	9	ACUTE DUODENAL ULCER WITH HEMORRHAGE WITHOUT MENTION OBSTRUCTION			
532.01	9	ACUTE DUODENAL ULCER WITH HEMORRHAGE AND OBSTRUCTION			
532.1	9	ACUTE DUODENAL ULCER WITH PERFORATION			
532.10	9	ACUTE DUODENAL ULCER WITH PERFORATION WITHOUT MENTION OBSTRUCTION			
532.11	9	ACUTE DUODENAL ULCER WITH PERFORATION AND OBSTRUCTION			
532.2	9	ACUTE DUODENAL ULCER WITH HEMORRHAGE AND PERFORATION			
532.20	9	ACUTE DUODENAL ULCER WITH HEMORRHAGE AND PERFORATION WITHOUT MENTION OBSTRUCTION			
532.21	9	ACUTE DUODENAL ULCER WITH HEMORRHAGE PERFORATION AND OBSTRUCTION			
532.3	9	ACUTE DUODENAL ULCER WITHOUT MENTION HEMORRHAGE/PERFORATION			
532.30	9	ACUTE DUODENAL ULCER WITHOUT MENTION HEMORRHAGE PERFORATION/OBSTRUCTION			
532.31	9	ACUTE DUODENAL ULCER WITHOUT MENTION HEMORRHAGE/PERFORATION WITH OBSTRUCTION			
532.4	9	CHRONIC/UNSPECIFIED DUODENAL ULCER WITH HEMORRHAGE			
532.40	9	CHRONIC/UNSPECIFIED DUODENAL ULCER WITH HEMORRHAGE WITHOUT MENTION OBSTRUCTION			
532.41	9	CHRONIC/UNSPECIFIED DUODENAL ULCER WITH HEMORRHAGE AND OBSTRUCTION			
532.5	9	CHRONIC/UNSPECIFIED DUODENAL ULCER WITH PERFORATION			
532.50	9	CHRONIC/UNSPECIFIED DUODENAL ULCER WITH PERFORATION WITHOUT MENTION OBSTRUCTION			
532.51	9	CHRONIC/UNSPECIFIED DUODENAL ULCER WITH PERFORATION AND OBSTRUCTION			
532.6	9	CHRONIC/UNSPECIFIED DUODENAL ULCER WITH HEMORRHAGE AND PERFORATION			

© 2021 Herrin J et al. *JAMA Network Open*.

532.60	9	CHRONIC / UNSPECIFIED DUOD ULCR WITH HEMORRHAGE AND PERFORATION WITHOUT OBSTRUCTION			
532.61	9	CHRONIC/UNSPECIFIED DUODENAL ULCER WITH HEMORRHAGE PERFORATION AND OBSTRUCTION			
533.0	9	ACUTE PEPTIC ULCER UNSPECIFIED SITE WITH HEMORRHAGE			
533.00	9	ACUTE PEPTC ULCER UNSPECIFIED SITE WITH HEMORRHAGE WITHOUT MENTION OBSTRUCTION			
533.01	9	ACUTE PEPTIC ULCER UNSPECIFIED SITE WITH HEMORRHAGE AND OBSTRUCTION			
533.1	9	ACUTE PEPTIC ULCER UNSPECIFIED SITE WITH PERFORATION			
533.10	9	ACUTE PEPTIC ULCER UNSPECIFIED SITE WITH PERFORATION WITHOUT MENTION OBSTRUCTION			
533.11	9	ACUTE PEPTIC ULCER UNSPECIFIED SITE WITH PERFORATION AND OBSTRUCTION			
533.2	9	ACUTE PEPTIC ULCER UNSPECIFIED SITE WITH HEMORRHAGE AND PERFORATION			
533.20	9	ACUTE PEPTC ULCER UNSPECIFIED SITE WITH HEMORRHAGE AND PERFORATION WITHOUT OBSTRUCTION			
533.21	9	ACUTE PEPTIC ULCER UNSPECIFIED SITE WITH HEMORRHAGE PERFORATION AND OBSTRUCTION			
533.3	9	ACUTE PEPTIC ULCER UNSPECIFIED SITE WITHOUT MENTION HEMORRHAGE AND PERFORATION			
533.30	9	ACUTE PEPTIC ULCER UNSPECIFIED SITE WITHOUT HEMORRHAGE PERFORATION/OBSTRUCTION			
533.31	9	ACUTE PEPTIC ULCER UNSPECIFIED SITE WITHOUT HEMORRHAGE AND PERFORATION WITH OBSTRUCTION			
533.4	9	CHRONIC/UNSPECIFIED PEPTIC ULCER UNSPECIFIED SITE WITH HEMORRHAGE			
533.40	9	CHRONIC/UNSPECIFIED PEPTIC ULCER UNSPECIFIED SITE WITH HEMORRHAGE WITHOUT OBSTRUCTION			
533.41	9	CHRONIC / UNSPECIFIED PEPTC ULCR UNSPECIFIED SITE WITH HEMORRHAGE AND OBSTRUCTION			
533.5	9	CHRONIC/UNSPECIFIED PEPTIC ULCER UNSPECIFIED SITE WITH PERFORATION			
533.50	9	CHRONIC / UNSPECIFIED PEPTC ULCR UNSPECIFIED SITE WITH PERFORATION WITHOUT OBSTRUCTION			
533.51	9	CHRONIC/UNSPECIFIED PEPTIC ULCER UNSPECIFIED SITE WITH PERFORATION AND OBSTRUCTION			
533.6	9	CHRONIC / UNSPECIFIED PEPTC ULCR UNSPECIFIED SITE WITH HEMORRHAGE AND PERFORATION			
533.60	9	CHRONIC/UNSPECIFIED PEPTIC ULCER WITH HEMORRHAGE AND PERFORATION WITHOUT OBSTRUCTION			
533.61	9	CHRONIC/UNSPECIFIED PEPTIC ULCER UNSPECIFIED SITE WITH HEMORRHAGE PERFORATION AND OBSTRUCTION			
534.0	9	ACUTE GASTROJEJUNAL ULCER WITH HEMORRHAGE			
534.00	9	ACUTE GASTROJEJUNAL ULCER WITH HEMORRHAGE WITHOUT MENTION OBSTRUCTION			
534.01	9	ACUTE GASTROJEJUNAL ULCER WITH HEMORRHAGE AND OBSTRUCTION			
534.1	9	ACUTE GASTROJEJUNAL ULCER WITH PERFORATION			
534.10	9	ACUTE GASTROJEJUNAL ULCER WITH PERFORATION WITHOUT MENTION OBSTRUCTION			
534.11	9	ACUTE GASTROJEJUNAL ULCER WITH PERFORATION AND OBSTRUCTION			
534.2	9	ACUTE GASTROJEJUNAL ULCER WITH HEMORRHAGE AND PERFORATION			
534.20	9	ACUTE GASTROJEJUNAL ULCER WITH HEMORRHAGE AND PERFORATION WITHOUT OBSTRUCTION			
534.21	9	ACUTE GASTROJEJUNAL ULCER WITH HEMORRHAGE PERFORATION AND OBSTRUCTION			
534.3	9	ACUTE GASTROJEJUNAL ULCER WITHOUT MENTION HEMORRHAGE PERFORATION			
534.30	9	ACUTE GASTROJEJUNAL ULCER WITHOUT MENTION HEMORRHAGE PERFORATION/OBSTRUCTION			
534.31	9	ACUTE GASTROJEJUNAL ULCER WITHOUT HEMORRHAGE/PERFORATION WITH OBSTRUCTION			
534.4	9	CHRONIC/UNSPECIFIED GASTROJEJUNAL ULCER WITH HEMORRHAGE			
534.40	9	CHRONIC/UNSPECIFIED GASTROJEJUNAL ULCER WITH HEMORRHAGE WITHOUT MENTION OBSTRUCTION			
534.41	9	CHRONIC/UNSPECIFIED GASTROJEJUNAL ULCER WITH HEMORRHAGE AND OBSTRUCTION			
534.5	9	CHRONIC/UNSPECIFIED GASTROJEJUNAL ULCER WITH PERFORATION			
534.50	9	CHRONIC/UNSPECIFIED GASTROJEJUNAL ULCER WITH PERFORATION WITHOUT MENTION OBSTRUCTION			
534.51	9	CHRONIC/UNSPECIFIED GASTROJEJUNAL ULCER WITH PERFORATION AND OBSTRUCTION			
534.6	9	CHRONIC/UNSPECIFIED GASTROJEJUNAL ULCER WITH HEMORRHAGE AND PERFORATION			
534.60	9	CHRONIC/UNSPECIFIED GASTROJEJUNAL ULCER WITH HEMORRHAGE AND PERFORATION WITHOUT OBSTRUCTION			
534.61	9	CHRONIC/UNSPECIFIED GASTROJEJUNAL ULCER WITH HEMORRHAGE PERFORATION AND OBSTRUCTION			
535.0	9	ACUTE GASTRITIS			
535.00	9	ACUTE GASTRITIS WITHOUT MENTION OF HEMORRHAGE			

© 2021 Herrin J et al. *JAMA Network Open*.

535.01	9	ACUTE GASTRITIS WITH HEMORRHAGE			
535.1	9	ATROPHIC GASTRITIS			
535.10	9	ATROPHIC GASTRITIS WITHOUT MENTION OF HEMORRHAGE			
535.11	9	ATROPHIC GASTRITIS WITH HEMORRHAGE			
535.2	9	GASTRIC MUCOSAL HYPERTROPHY			
535.20	9	GASTRIC MUCOS HYPERTROPHY WITHOUT MENTION HEMORRHAGE			
535.21	9	GASTRIC MUCOSAL HYPERTROPHY WITH HEMORRHAGE			
535.3	9	ALCOHOLIC GASTRITIS			
535.30	9	ALCOHOLIC GASTRITIS WITHOUT MENTION HEMORRHAGE			
535.31	9	ALCOHOLIC GASTRITIS WITH HEMORRHAGE			
535.4	9	OTHER SPECIFIED GASTRITIS			
535.40	9	OTHER SPECIFIED GASTRITIS WITHOUT MENTION HEMORRHAGE			
535.41	9	OTHER SPECIFIED GASTRITIS WITH HEMORRHAGE			
535.5	9	UNSPECIFIED GASTRITIS AND GASTRODUODENITIS			
535.50	9	UNSPECIFIED GASTRITIS AND GASTRODUODITIS WITHOUT MENTION HEMORRHAGE			
535.51	9	UNSPECIFIED GASTRITIS AND GASTRODUODENITIS WITH HEMORRHAGE			
535.6	9	DUODENITIS			
535.60	9	DUODENITIS WITHOUT MENTION OF HEMORRHAGE			
535.61	9	DUODENITIS WITH HEMORRHAGE			
537.83	9	ANGIODYSPLASIA OF STOMACH AND DUODENUM WITH HEMORRHAGE			
537.84	9	DIEULAFOY LESION OF STOMACH AND DUODENUM			
578.	9	GASTROINTESTINAL HEMORRHAGE			
K22.6	10	GASTRO-ESOPHAGEAL LACERATION-HEMORRHAGE SYNDROME			
K25.0	10	ACUTE GASTRIC ULCER WITH HEMORRHAGE			
K25.1	10	ACUTE GASTRIC ULCER WITH PERFORATION			
K25.2	10	ACUTE GASTRIC ULCER WITH BOTH HEMORRHAGE AND PERFORATION			
K25.3	10	ACUTE GASTRIC ULCER WITHOUT HEMORRHAGE OR PERFORATION			
K25.4	10	CHRONIC OR UNSPECIFIED GASTRIC ULCER WITH HEMORRHAGE			
K25.5	10	CHRONIC OR UNSPECIFIED GASTRIC ULCER WITH PERFORATION			
K25.6	10	CHRONIC OR UNSPECIFIED GASTRIC ULCER WITH BOTH HEMORRHAGE AND PERFORATION			
K26.0	10	ACUTE DUODENAL ULCER WITH HEMORRHAGE			
K26.1	10	ACUTE DUODENAL ULCER WITH PERFORATION			
K26.2	10	ACUTE DUODENAL ULCER WITH BOTH HEMORRHAGE AND PERFORATION			
K26.3	10	ACUTE DUODENAL ULCER WITHOUT HEMORRHAGE OR PERFORATION			
K26.4	10	CHRONIC OR UNSPECIFIED DUODENAL ULCER WITH HEMORRHAGE			
K26.5	10	CHRONIC OR UNSPECIFIED DUODENAL ULCER WITH PERFORATION			
K26.6	10	CHRONIC OR UNSPECIFIED DUODENAL ULCER WITH BOTH HEMORRHAGE AND PERFORATION			
K27.0	10	ACUTE PEPTIC ULCER SITE UNSPECIFIED WITH HEMORRHAGE			
K27.1	10	ACUTE PEPTIC ULCER SITE UNSPECIFIED WITH PERFORATION			
K27.2	10	ACUTE PEPTIC ULCER SITE UNSPECIFIED WITH BOTH HEMORRHAGE AND PERFORATION			
K27.3	10	ACUTE PEPTIC ULCER SITE UNSPECIFIED WITHOUT HEMORRHAGE OR PERFORATION			
K27.4	10	CHRONIC OR UNSPECIFIED PEPTIC ULCER SITE UNSPECIFIED WITH HEMORRHAGE			
K27.5	10	CHRONIC OR UNSPECIFIED PEPTIC ULCER SITE UNSPECIFIED WITH PERFORATION			
K27.6	10	CHRONIC OR UNSPECIFIED PEPTIC ULCER SITE UNSPECIFIED WITH BOTH HEMORRHAGE AND PERFORATION			
K28.0	10	ACUTE GASTROJEJUNAL ULCER WITH HEMORRHAGE			
K28.1	10	ACUTE GASTROJEJUNAL ULCER WITH PERFORATION			
K28.2	10	ACUTE GASTROJEJUNAL ULCER WITH BOTH HEMORRHAGE AND PERFORATION			
K28.3	10	ACUTE GASTROJEJUNAL ULCER WITHOUT HEMORRHAGE OR PERFORATION			
K28.4	10	CHRONIC OR UNSPECIFIED GASTROJEJUNAL ULCER WITH HEMORRHAGE			
K28.5	10	CHRONIC OR UNSPECIFIED GASTROJEJUNAL ULCER WITH PERFORATION			
K28.6	10	CHRONIC OR UNSPECIFIED GASTROJEJUNAL ULCER WITH BOTH HEMORRHAGE AND PERFORATION			
K29.00	10	ACUTE GASTRITIS WITHOUT BLEEDING			
K29.01	10	ACUTE GASTRITIS WITH BLEEDING			
K29.30	10	CHRONIC SUPERFICIAL GASTRITIS WITHOUT BLEEDING			
K29.31	10	CHRONIC SUPERFICIAL GASTRITIS WITH BLEEDING			
K29.40	10	CHRONIC ATROPHIC GASTRITIS WITHOUT BLEEDING			
K29.41	10	CHRONIC ATROPHIC GASTRITIS WITH BLEEDING			
K29.50	10	UNSPECIFIED CHRONIC GASTRITIS WITHOUT BLEEDING			
K29.51	10	UNSPECIFIED CHRONIC GASTRITIS WITH BLEEDING			
K29.60	10	OTHER GASTRITIS WITHOUT BLEEDING			
K29.61	10	OTHER GASTRITIS WITH BLEEDING			
K29.70	10	GASTRITIS UNSPECIFIED WITHOUT BLEEDING			
K29.71	10	GASTRITIS UNSPECIFIED WITH BLEEDING			
K29.80	10	DUODENITIS WITHOUT BLEEDING			
K29.81	10	DUODENITIS WITH BLEEDING			
K29.90	10	GASTRODUODENITIS UNSPECIFIED WITHOUT BLEEDING			
K29.91	10	GASTRODUODENITIS UNSPECIFIED WITH BLEEDING			
K31.811	10	ANGIODYSPLASIA STOMACH AND DUODENUM WITH BLEEDING			
K31.82	10	DIEULAFOY LESION HEMORRHAGIC STOMACH AND DUODENUM			

© 2021 Herrin J et al. *JAMA Network Open*.

eTable 2. Performance results for classification models.

method	Time (months)	AUC	sensitivity	specificity	Pos Pred Value	Neg Pred Value	Balanced Accuracy	threshold	data
RegCox	6	0.68(0.67,0.71)	0.54(0.50,0.58)	0.72(0.71,0.72)	0.05(0.05,0.06)	0.98(0.98,0.98)	0.63(0.61,0.65)	0.03(0.03,0.03)	internal
RegCox	12	0.67(0.66,0.68)	0.55(0.53,0.57)	0.69(0.68,0.70)	0.07(0.06,0.07)	0.97(0.97,0.98)	0.62(0.61,0.63)	0.04(0.04,0.04)	internal
RegCox	6	0.68	0.55	0.70	0.04	0.99	0.63	0.03	external
RegCox	12	0.67	0.56	0.67	0.04	0.99	0.62	0.04	external
RSF	6	0.68(0.67,0.71)	0.59(0.57,0.63)	0.67(0.67,0.68)	0.05(0.05,0.05)	0.98(0.98,0.98)	0.63(0.62,0.65)	0.03(0.03,0.03)	internal
RSF	12	0.67(0.66,0.68)	0.61(0.58,0.64)	0.63(0.62,0.63)	0.06(0.06,0.06)	0.98(0.97,0.98)	0.62(0.61,0.64)	0.04(0.04,0.04)	internal
RSF	6	0.67	0.58	0.65	0.03	0.99	0.62	0.03	external
RSF	12	0.66	0.62	0.6	0.03	0.99	0.61	0.04	external
XGBoost	6	0.69(0.67,0.71)	0.56(0.52,0.60)	0.71(0.71,0.71)	0.05(0.05,0.05)	0.98(0.98,0.98)	0.64(0.62,0.65)	0.03(0.03,0.03)	internal
XGBoost	12	0.67(0.66,0.69)	0.57(0.54,0.61)	0.68(0.68,0.69)	0.07(0.06,0.07)	0.98(0.97,0.98)	0.62(0.61,0.64)	0.04(0.04,0.04)	internal
XGBoost	6	0.68	0.56	0.70	0.03	0.99	0.63	0.03	external
XGBoost	12	0.67	0.57	0.67	0.04	0.99	0.62	0.04	external
HASBLED	6	0.61(0.59,0.62)	0.54(0.51,0.56)	0.62(0.62,0.63)	0.03(0.03,0.04)	0.98(0.98,0.99)	0.58(0.56,0.59)	3	internal
HASBLED	12	0.60(0.59,0.61)	0.53(0.51,0.54)	0.62(0.62,0.63)	0.05(0.04,0.05)	0.98(0.97,0.98)	0.57(0.56,0.58)	3	internal
HASBLED	6	0.60	0.57	0.58	0.02	0.99	0.56	3	external
HASBLED	12	0.59	0.56	0.58	0.03	0.99	0.56	3	external

© 2021 Herrin J et al. *JAMA Network Open*.

eTable 3. Performance of time-to-event models, developed and validated using only patients with AF.

method	Time (months)	AUC	sensitivity	specificity	Pos Pred Value	Neg Pred Value	Balanced Accuracy	threshold	data
XGBoost	6	0.66(0.64,0.69)	0.61(0.20,0.99)	0.55(0.05,0.90)	0.05(0.03,0.06)	0.98(0.97,0.99)	0.58(0.52,0.62)	0.02(0.02,0.02)	internal
XGBoost	12	0.65(0.63,0.67)	0.65(0.36,0.92)	0.53(0.21,0.81)	0.06(0.05,0.08)	0.97(0.97,0.98)	0.59(0.56,0.61)	0.04(0.04,0.04)	internal
XGBoost	6	0.66	0.63	0.59	0.03	0.99	0.61	0.02	external
XGBoost	12	0.65	0.69	0.5	0.03	0.99	0.6	0.04	external
RSF	6	0.63(0.59,0.67)	0.03(0.00,0.07)	0.99(0.97,1.00)	0.07(0.00,0.16)	0.97(0.97,0.97)	0.51(0.50,0.52)	0.17(0.17,0.17)	internal
RSF	12	0.63(0.58,0.65)	0.04(0.00,0.09)	0.98(0.95,1.00)	0.09(0.04,0.12)	0.96(0.96,0.96)	0.51(0.50,0.52)	0.21(0.21,0.21)	internal
RSF	6	0.61	0.06	0.97	0.04	0.98	0.52	0.17	external
RSF	12	0.6	0.08	0.95	0.04	0.98	0.52	0.21	external
RegCox	6	0.67(0.64,0.69)	0.56(0.51,0.60)	0.68(0.67,0.68)	0.05(0.05,0.06)	0.98(0.98,0.98)	0.62(0.59,0.64)	0.04(0.04,0.04)	internal
RegCox	12	0.66(0.63,0.67)	0.61(0.57,0.64)	0.61(0.59,0.62)	0.06(0.06,0.07)	0.97(0.97,0.97)	0.61(0.59,0.62)	0.06(0.06,0.06)	internal
RegCox	6	0.67	0.57	0.67	0.04	0.99	0.62	0.04	external
RegCox	12	0.66	0.63	0.59	0.04	0.99	0.61	0.06	external
HASBLED	6	0.61(0.57,0.64)	0.50(0.45,0.56)	0.65(0.64,0.65)	0.04(0.04,0.04)	0.98(0.98,0.99)	0.58(0.55,0.59)	3	internal
HASBLED	12	0.60(0.58,0.62)	0.48(0.46,0.52)	0.65(0.64,0.65)	0.05(0.05,0.05)	0.97(0.97,0.98)	0.57(0.56,0.59)	3	internal
HASBLED	6	0.6	0.53	0.61	0.02	0.99	0.56	3	external
HASBLED	12	0.59	0.51	0.61	0.03	0.98	0.56	3	external

© 2021 Herrin J et al. *JAMA Network Open*.
